# The Roles of E93 and Kr-h1 in Metamorphosis of *Nilaparvata lugens*

**DOI:** 10.3389/fphys.2018.01677

**Published:** 2018-11-22

**Authors:** Kai Long Li, San Yue Yuan, Satyabrata Nanda, Wei Xia Wang, Feng Xiang Lai, Qiang Fu, Pin Jun Wan

**Affiliations:** ^1^State Key Laboratory of Rice Biology, China National Rice Research Institute, Chinese Academy of Agricultural Sciences, Hangzhou, China; ^2^Hunan Institute of Food Quality Supervision Inspection and Research, Changsha, China

**Keywords:** E93, Kr-h1, *Nilaparvata lugens*, metamorphosis, RNAi

## Abstract

Metamorphosis is a crucial process in insect development. Ecdysone-induced protein 93 (E93) is a determinant that promotes adult metamorphosis in both hemimetabolous and holometabolous insects. Krüppel-homolog 1 (Kr-h1), an early juvenile hormone (JH)-inducible gene, participates in JH signaling pathway controlling insect metamorphosis. In the current study, an *E93* cDNA (*NlE93*) and two *Kr-h1* cDNA variants (*NlKr-h1-a* and *NlKr-h1-b*) were cloned from *Nilaparvata lugens* (Stål), one of the most destructive hemimetabolous insect pests on rice. Multiple sequence alignment showed that both NlE93 and NlKr-h1 share high identity with their orthologs from other insects. The expression patterns revealed that decreasing *NlKr-h1* mRNA levels were correlated with increasing *NlE93* mRNA levels and vice versa. Moreover, RNA interference (RNAi) assays showed that the knockdown of one of the two genes resulted in significantly upregulated expression of the other. Correspondingly, phenotypical observation of the RNAi insects revealed that depletion of *NlE93* prevented nymph–adult transition (causing a supernumerary nymphal instar), while depletion of *NlKr-h1* triggered precocious formation of incomplete adult features. The results suggest that *Nlkr-h1* and *NlE93* are mutual repressors, fitting into the MEKRE93 pathway. The balance between these two genes plays a critical role in the metamorphosis of *N. lugens* determining the proper timing for activating metamorphosis during the nymphal stage.

## Introduction

The brown planthopper, *Nilaparvata lugens* (Stål), is one of the most destructive pests in rice. The insect feeds directly from the phloem tissues of rice plants causing rapid wilting or drying of the crop, which is referred as “hopperburn” (Cheng et al., 2003; Heong and Hardy, 2009). As a typical hemimetabolous insect, *N. lugens* undergoes four immature molts of the five nymphal instars, followed by a nymph–adult metamorphic molt to adults with wings and external genitals for reproduction. These processes are controlled by two important hormones, juvenile hormone (JH) and ecdysone (E) (Jindra et al., 2013). JH plays a crucial role ensuring the growth of the immatures and regulating metamorphosis (Truman and Riddiford, 2002; Riddiford, 2008). Previous studies showed that Krüppel homolog 1 (Kr-h1) was involved in the JH anti-metamorphic action in hemimetabolous insects as an important JH transducer (Konopova et al., 2011; Lozano and Belles, 2011). Kr-h1 is a C2H2 zinc-finger type transcription factor that has been identified as a JH early-inducible gene and involved in many aspects of insect physiology, such as development (Miyakawa et al., 2018), metamorphosis (Minakuchi et al., 2008, 2009; Konopova et al., 2011; Lozano and Belles, 2011; Kayukawa et al., 2012, 2014) and reproduction (Yue et al., 2018). *Kr-h1* is activated by the interaction between the JH response element located in the 5′ upstream of *Kr-h1* and the JH/Met/Tai complex consisting of JH, JH receptor methoprene tolerant (Met), and a bHLH-PAS transcription factor Taiman (Tai). Therefore, JH induced *Kr-h1* works downstream of Met and Tai (Kayukawa et al., 2012). The knockdown of *Kr-h1* repressed transitions of nymph to adult or larva to adult, and caused the precocious metamorphosis (Minakuchi et al., 2009; Konopova et al., 2011; Lozano and Belles, 2011). Previous studies reported that *Kr-h1* repress metamorphosis via suppression of ecdysone-inducible genes such as *Broad* in *Tribolium* *castaneum* (Minakuchi et al., 2009) or *Drosophila melanogaster* (Huang et al., 2011) and ecdysone-induced protein 93 (E93) in *Blattella germanica* (Belles and Santos, 2014; Ureña et al., 2014) or *Bombyx mori* (Kayukawa et al., 2017). Functional analysis of a previously identified *Kr-h1* transcript in *N. lugens* revealed that *Kr-h1* could affect wing and external genitalia development in both males and females (Jin et al., 2014; Jiang et al., 2017). Moreover, a recent study revealed that *Kr-h1* plays a critical role in early development of scale insects independent from the JH signaling pathway (Vea et al., 2016).

The E93, a helix-turn-helix (HTH) transcription factor acting as a universal adult specifier, is a determinant of adult metamorphosis in both hemimetabolous and holometabolous insects (Ureña et al., 2014). The metamorphosis process involves regulating cell death of nymph/larva tissues and morphogenesis of adult tissues (Buszczak and Segraves, 2000). Several studies in *Drosophila* flies demonstrated that E93 plays a role in transducing 20-hydroxyecdysone (20E) signaling to induce programmed cell death in the midgut (Lee et al., 2002a), salivary gland (Lee et al., 2002b), and fat body (Liu et al., 2014), and causes the remodeling of those tissues during metamorphosis. Moreover, E93 has shown to be a key factor that triggers metamorphosis, and repress the expression of the JH-induced *Kr-h1* (Belles and Santos, 2014) and the ecdysone-induced Broad (Ureña et al., 2014). Importantly, *Kr-h1* represses the action of *E93* in the context of the MEKRE93 pathway (Belles and Santos, 2014), which is universal in metamorphosing insects. According to this pathway, metamorphosis is mediated by a decrease of JH that triggers a downregulation of *Kr-h1*, which, in turn, de-represses *E93* expression (Belles and Santos, 2014). This shows, therefore, that Kr-h1 and E93 are fundamental players in the regulation of metamorphosis in insects.

In the present study, we have cloned the *E93* and *Kr-h1* cDNAs from *N. lugens* and characterized their developmental and tissue-specific expression profiles. Furthermore, gene knockdowns were performed by *NlE93*-dsRNA and *NlKr-h1*-dsRNA injections to observe the effect of RNA interference (RNAi) on the metamorphosis of *N. lugens*. The results add to the overall understanding of the gene functionalities, and provide more insights in elucidating the insect metamorphosis regulations.

## Materials and Methods

### Insects

*Nilaparvata lugens* colonies were obtained from a local rice field near China National Rice Research Institute, Hangzhou, China (119.93°N, 30.08°E), and reared on rice (*Oryza sativa*) variety Taichung Native 1 (TN1, susceptible to almost all herbivores on rice) at 27 ± 0.5°C and 75 ± 5% relative humidity under a 16/8 h light/dark photoperiod.

### Molecular Cloning and Sequence Analysis

Based on the published *N. lugens* genome (accession No. GCA_000757685.1) and transcriptome (accession No. SRX326774) (Xue et al., 2014; Wan et al., 2015), *E93* and *Kr-h1* homologs were identified and their sequences were confirmed by reverse transcription polymerase chain reaction (RT-PCR) using primers listed in Supplementary Table S1. Total RNA was extracted with the Trizol Total RNA Isolation Kit (Invitrogen, Carlsbad, CA, United States) according to the manufacturer’s instructions. The RNA concentration and purity were measured with a NanoDrop 1000 spectrophotometer (Thermo Fisher Scientific, Rockford, IL, United States) and the integrity was checked by agarose gel electrophoresis. A quantity of 1 μg of the total RNA was reverse transcribed to cDNA by using the ReverTra Ace qPCR RT Kit (Toyobo. Co. Ltd., Osaka, Japan) following the manufacturer’s manual. The PCR product was gel purified, ligated into the vector TOPO2.1 (Invitrogen) and transformed into *Escherichia coli* DH5α competent cells (Novagen, Darmstadt, Germany). The recombinant plasmids from 10 independent subclones were fully sequenced on an Applied Biosystems 3730 automated sequencer (Foster City, CA, United States) from both directions.

E93 and Kr-h1 from *D. melanogaster, Culex quinquefasciatus, Aedes aegypti, B. mori, Apis mellifera, T. castaneum, Frankliniella occidentalis, B. germanica, Reticulitermes speratus*, and *Rhodnius prolixus* were aligned with that from *N. lugens* using ClustalW2 (Larkin et al., 2007), respectively. The conserved domains of putative E93 and Kr-hl were predicted by using SMART (Letunic and Bork, 2018) and InterPro (Finn et al., 2017).

### Quantitative Real-Time PCR (qRT-PCR)

Total RNA samples were isolated from eggs, whole bodies (from 20 to 50 individuals) of the first- through third-instar nymphs (N1, N2, and N3), fourth- and fifth-instar nymphs at a time interval of 1 day (N4D1, N4D2, N4D3, N5D1, N5D2, N5D3, and N5D4), newly emerged female adults (New-A, short-winged), and 2-day-old female adults (AD2, short-winged), and from head (He), thorax (Th), abdomen (Ab), integument (In), wingbud (Wi), midgut (Mg), leg (Lg), and fat body (Fb) of 50 fourth-instar nymphs. The recommended stably expressed reference genes *ribosomal protein S15e* (*rps15*) and *rps11* (primers listed in Supplementary Table S1) were used as internal control genes (Yuan et al., 2014). A RT negative control (without reverse transcriptase) and a non-template negative control were included for each primer set to confirm the absence of genomic DNA and to check for primer-dimer or contamination in the reactions, respectively. All experiments were replicated three times and each sample was repeated in technical triplicates. The transcriptional levels of the target genes were calculated by the 2^-ΔΔCT^ method (Livak and Schmittgen, 2001), using the geometric mean of internal control genes for normalization. All methods and data were conformed to the MIQE guidelines (Bustin et al., 2009).

### dsRNA Synthesis and Bioassay

Two dsDNA fragments (ds*NlE93* and ds*NlKr-h1*) and a green fluorescent protein (ds*GFP*) fragment were amplified by PCR using specific primers (Supplementary Table S1) conjugated with the T7 RNA polymerase promoter (5′-taatacgactcactataggg-3′). The PCR products were gel purified and used as templates to synthesize dsRNA using MEGAscript T7 High Yield Transcription Kit (Ambion, Austin, TX, United States). The quality and concentration of the dsRNA were determined by agarose gel electrophoresis and the Nanodrop 1000 spectrophotometer and kept at -80°C until use. The dsRNA of green fluorescent protein (ds*GFP*) was used as a negative control for any non-specific effects of dsRNA.

RNAi bioassay was performed by injection as previously reported (Wang et al., 2018). Briefly, 200 ng (0.05 μL) and 400 ng (0.1 μL) dsRNA (concentration estimated 4.000 mg/mL) and the same amount of ds*GFP* (negative control) were injected into the newly emerged fourth- and fifth-instar nymphs (N4D1 and N5D1; Xu et al., 2015). Survival rates and individual morphological phenotypes were recorded. Abnormal phenotypes were dissected for further observation. A total of 200 nymphs (10 replicates, 20 individuals in each replicate) were used for each treatment. Three replicates were used for survival evaluation, three replicates for phenotypic evaluation, three replicates for qRT-PCR, and one replicate was kept as a backup replication. To confirm RNAi, at 4–6 days after injection, total RNA was isolated from 15 individuals to check the transcript levels of the target genes by qRT-PCR. Three biological replicates and three technical replicates for each biological replicate were included for each experiment.

### Data Analyses

Data analysis was carried out using Data Processing System software (Tang and Zhang, 2013). The student’s *t*-test was applied for comparisons of two samples and one-way analysis of variance (ANOVA) with the Tukey’s test were applied for comparing the differences among three or more samples.

## Results

### Identification of *E93* and *Kr-h1* Genes in *N. lugens*

The cDNA of *E93* gene in *N. lugens* was cloned and sequenced, and the sequence was submitted to GenBank (KU194468). The gene contained complete coding regions encoding 1048 amino acid residues. In the meanwhile, two transcript variants of *Kr-h1* gene (*NlKr-h1-a* and *NlKr-h1-b*) were cloned, which contained complete coding regions encoding 610 and 591 amino acid residues, respectively. *NlKr-h1-a* was found to be identical with the already reported *NlKr-h1* of *N. lugens* (Jin et al., 2014), whereas the second transcript variant, *NlKr-h1-b* (KT936461), was longer in length containing additional 5′- and 3′-UTRs. Furthermore, the protein-coding regions of *NlKr-h1-a* and *NlKr-h1-b* were found to be identical.

Sequence alignments showed that NlE93 shared the highest identity with that of *T. castaneum* (46.0%), followed by 43.6–18.0% identities with that of *B. germanica, B. mori* and *D. melanogaster*. NlKr-h1-b shared the highest identity with that of *R. prolixus* (68.1%), followed by 62.9–39.9% identities with that of *B. germanica, T. castaneum, A. mellifera, B. mori, A. aegypti, C. quinquefasciatus*, and *D. melanogaster* (Supplementary Figure S1B).

**FIGURE 1:**
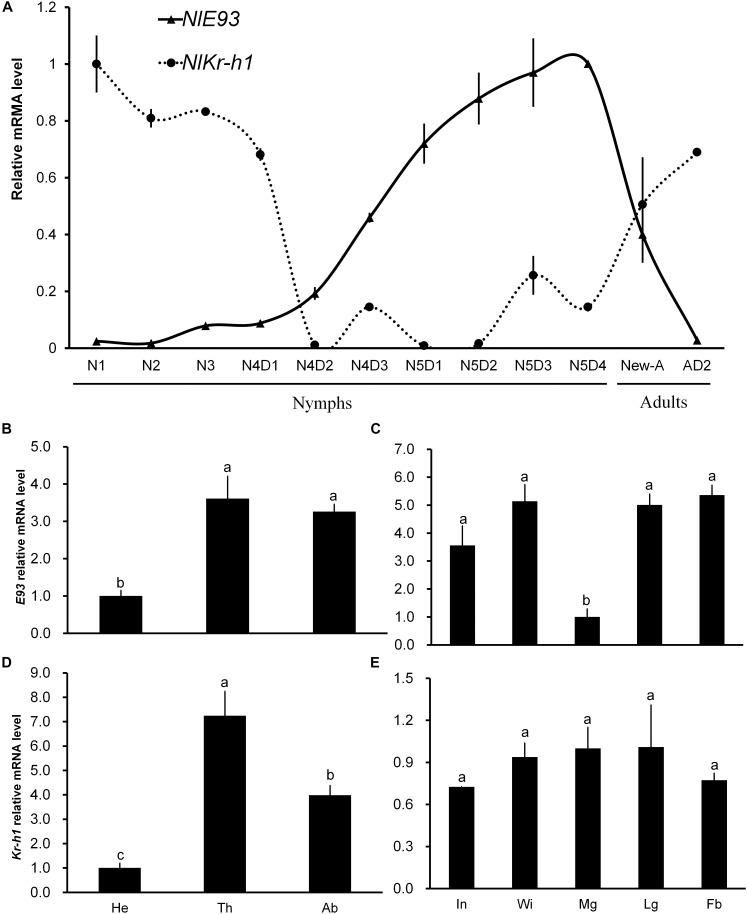
Temporal and spatial expression profiles of *NlE93* and *NlKr-h1.* **(A)** Relative expression level of *NlE93* and *NlE93* at different developmental stages. The whole bodies of 20–50 individuals of N1 (the first-instar nymph) to N5D4 (day 4 of the fifth-instar nymph), New-A (newly emerged female adults), Temporal and AD2 (2-day-old female adults) were collected and pooled to be used for total RNA isolation. **(B,C)** *NlE93* relative expression levels of head (He), thorax (Th), abdomen (Ab), integument (In), wingbud (Wi), midgut (Mg), leg (Lg), and fat body (Fb) of fourth and fifth instar of nymphs. **(D,E)** *Nlkr-h1* relative expression levels of head (He), thorax (Th), abdomen (Ab), integument (In), wingbud (Wi), midgut (Mg), leg (Lg), and fat body (Fb) of fourth- and fifth-instar nymphs. The various body parts were dissected from 50 to 100 individuals, and were pooled for the measurements. The relative expressions were 2^-ΔΔCT^ values (±SE) normalized to the geometrical mean of two housekeeping gene expressions. SE was determined from three independent biological replicates, each with three technical replications. Different letters indicate a significant difference (one-way ANOVA) at *P*-value < 0.05.

E93 proteins from *N. lugens* and other insects contain a highly conserved psq-type HTH domain that is constituted of three-α-helix structure in the C-terminus (Supplementary Figure S1A). The two NlKr-h1 proteins contained eight C2H2-type Zinc finger motifs which are constituted of a short beta hairpin and an alpha helix (beta/beta/alpha structure; Figure 1B), respectively. The comparisons of these two proteins to that of other insects revealed that the interval amino acid lengths of the first and second Zinc finger motifs are diverged. For instance, the interval length is eight or nine amino acid residues in most insects, but in dipterans, this length is much longer, e.g., 34, 37, and 56 amino acids in *A. aegypti, C. quinquefasciatus*, and *D. melanogaster*, respectively. Although, the “A” (LP(L/P)RKR) and “B” (RX_2_SVIX_2_A) motifs in the C-terminus are conserved, the interval amino acid lengths are the attributes that are distinct among the insect Kr-hl orthologs (Shpigler et al., 2010). Additionally, the length of the interval amino acid residues is much longer in the dipteran lineage as compared to other orders (Supplementary Figure S1B).

### Expression Profiles of *NlE93* and *NlKr-h1*

*NlE93* transcription of different developmental stages showed that the transcript level started to increase in the 2-day-old fourth-instar nymphs (N4D2), reached its peak in the 4-day-old fifth-instar nymphs (N5D4), and then declined in adults. The *NlKr-h1* transcript level was stable from N1 to early N4 (N4D1), and then declined rapidly in N4D2. *NlKr-h1* exhibited two small expression peaks during the late stage of fourth and fifth instar periods, and continued to increase gradually as adult aged (Figure 1A). This profile was contrary to that of *NlE93*.

The spatial expression profiles of *NlE93* and *NlKr-h1* in the fourth- and fifth-instar nymphs were analyzed (Figures 1B–E). Both *NlE93* and *NlKr-h1* were expressed at the lowest level in the head compared to the thorax and abdomen. *NlE93* expression was found to be similar in thorax and abdomen, whereas *NlKr-h1* was expressed higher in the thorax than in the abdomen (Figures 1B,D). *NlE93* expression in midgut was significantly lower than that of in integument (In), wingbud (Wi), leg (Lg), and fat body (Fb), while *NlKr-h1* expressed similarly in all these tissues (Figures 1C,E).

### Depletion of NlE93 Prevented Nymph–Adult Transition

**FIGURE 2:**
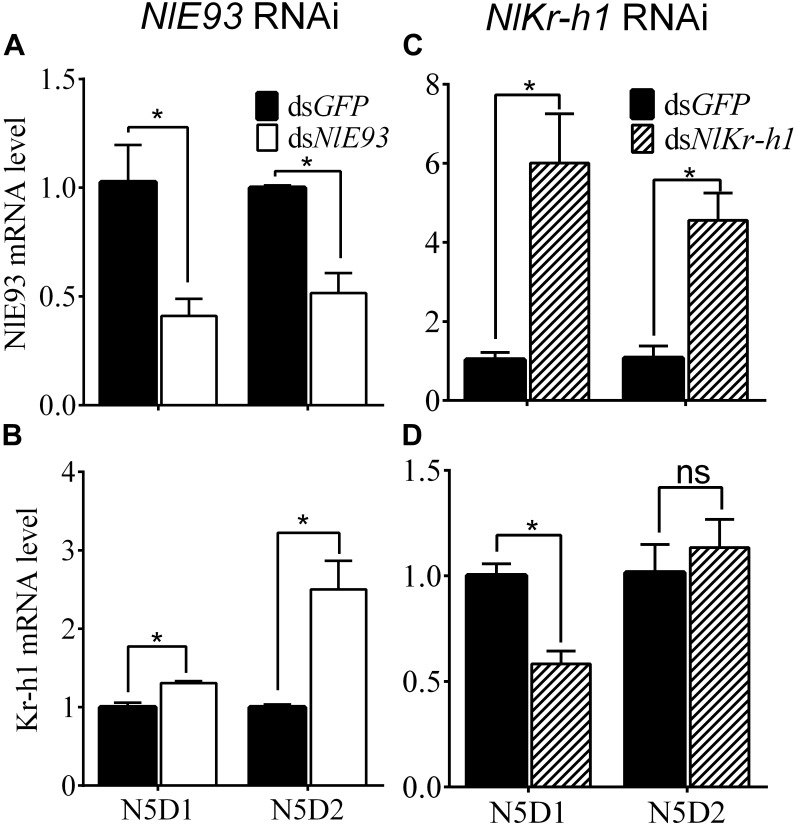
Effect of ds*NlE93* **(A,B)** or ds*NlKr-h1* **(C,D)** on the expression levels of *NlE93* and *NlKr-h1* of nymphs. The bars represent 2^-ΔΔCT^ values (±SE) normalized to the geometrical mean of the housekeeping gene expression. SE was determined from three independent biological replicates, each with three technical replications. ^∗^ indicates a significant difference at *P*-value < 0.05 (*t*-test).

The psq-type HTH domain region of *NlE93* was designed for dsRNA synthesis. *NlE93* dsRNA injection (200 ng) into newly emerged fourth-instar nymphs (N4D1) resulted in significantly lower *NlE93* transcript levels in N5D1 and N5D2. The expression of *NlE93* was reduced by 60.0% (*P* = 0.0308) and 48.4% (*P* = 0.0065) in N5D1 and N5D2, respectively, compared to the ds*GFP* controls (Figure 2A), whereas *NlKr-h1* mRNA levels were significantly upregulated by 1.3 folds (*P* = 0.0074) and 2.5 folds (*P* = 0.0149), respectively (Figure 2B).

**FIGURE 3:**
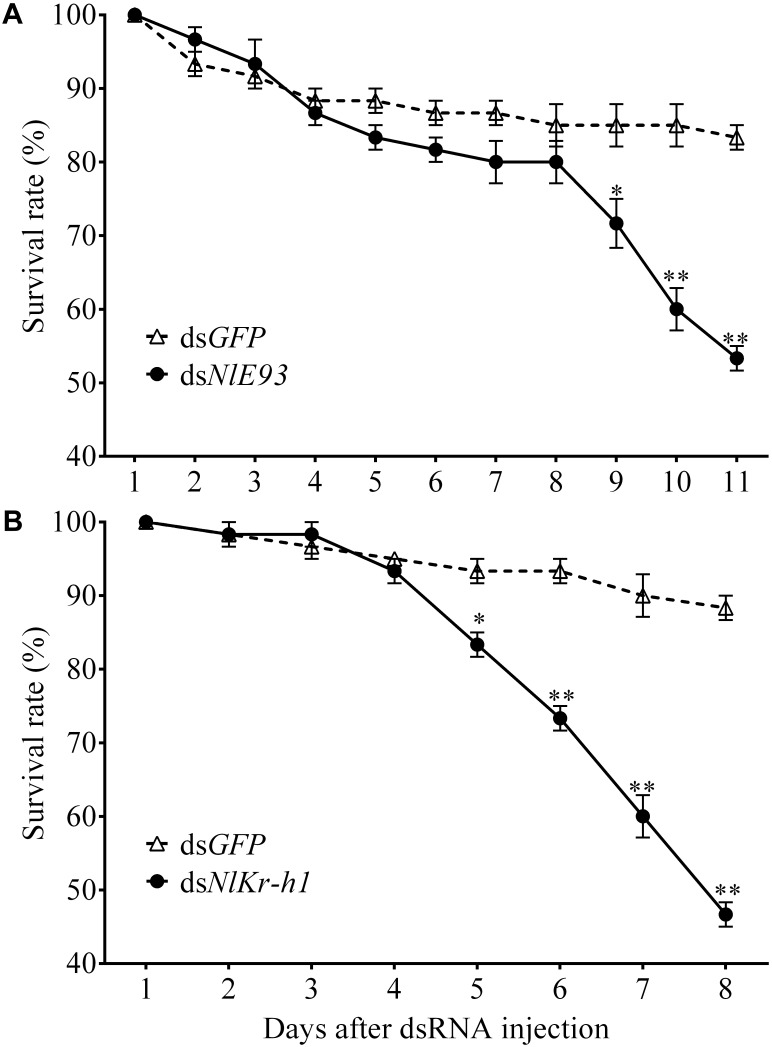
The survival rate of *N. lugens* subjected to ds*NlE93* **(A)** and ds*NlKr-h1* **(B)** injection. The survive rate of the *NlE93*i treatment significantly declined after the supernumerary N6 instar compared to the ds*GFP* control. The *Nlkr-h1* treatment caused significant survival declines at 5 days after injection and beyond. ^∗^ and ^∗∗^ indicate a significant difference at *P*-value < 0.05 or <0.01 (*t*-test) from the untreated.

**FIGURE 4:**
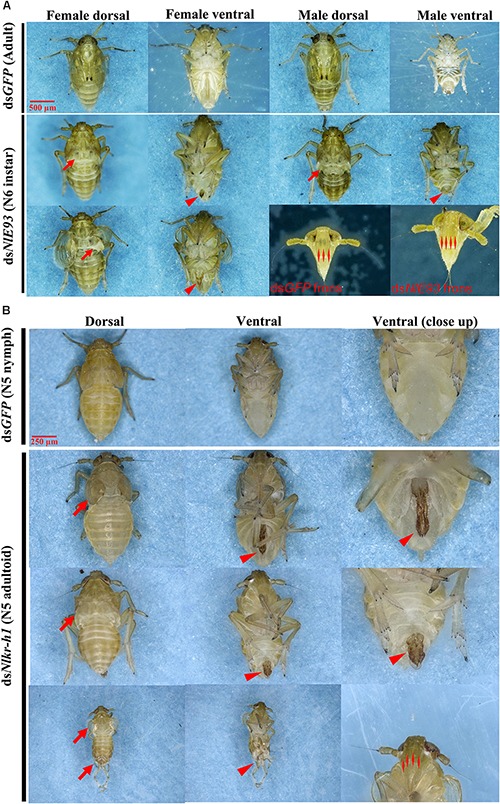
The phenotypes of *N. lugens* subjected to ds*NlE93* and ds*NlKr-h1* injection. **(A)** The abnormal phenotype of *N. lugens* subjected to ds*NlE93* injection in the fourth-instar nymphs of 1-day old. The resulted supernumerary N6 instar nymphs had no fully developed wings or no wings at all (denoted by arrows), no mature external genitalia (denoted with a triangle), and four carinae on the frons of adult features (denoted with double triangles). The ds*GFP* injected nymphs successfully molted to normal adults. **(B)** The abnormal phenotype of *N. lugens* subjected to ds*NlKr-h1* injection in the fourth instar nymphs of 1-day old. The resulted individuals were precocious nymph–adult intermediates (N5 adultoid) with no fully extended membranous wings (denoted by arrows) and deformed external genitalia rudiment (denoted with a triangle). The ds*GFP* injected nymphs successfully molted to normal N5 nymphs.

*NlE93* RNAi resulted in significantly higher mortality (*P* = 0.039), which started 9 days after injection (Figure 3A). At the phenotypic level, the ds*GFP* group (*n* = 51 out of 60) successfully molted to N5 nymphs and then to normal adults, while the *NlE93* dsRNA treated individuals (*n* = 49) molted to normal N5 nymphs, but most of them (*n* = 45) failed to metamorphose into adults, instead they repeated the nymphal molt to a deformed supernumerary N6 instar that lacked typical adult features such as fully developed wings, mature external genitalia, and three carinae on the frons (Figure 4A). Furthermore, the N6 *NlE93i* individuals could not continuously molt to another supernumerary instar and suffered continuous mortality, though new cuticle was formed under the old (duplicated cuticle structures; Supplementary Figure S2).

### Depletion of NlKr-h1 Triggered Precocious Formation of Adult Features

The C2H2-type Zinc finger motif region of *NlKr-h1* was designed for dsRNA synthesis, and this dsRNA was able to act on both identified transcript variants of *NlKr-h1*. Injection of 100 ng of *NlKr-h1* dsRNA into the fourth-instar nymphs significantly reduced *NlKr-h1* transcript levels by 41.8% (*P* = 0.0068) in 1-day-old fifty-instar nymphs (N5D1) compared to the ds*GFP* controls. No significant difference was found (*P* = 0.5658) in 2-day-old 50 instar nymphs (N5D2) between ds*NlKr-h1*-treated and the control samples (Figure 2C). Interestingly, when the ds*NlKr-h1* was injected into the fifth-instar nymphs, no difference in the expression level was detected between ds*NlKr-h1*-treated and ds*GFP* controls (data not shown, *P* = 0.4561). Conversely, *NlE93* mRNA levels were significantly upregulated by 5.8 folds (*P* = 0.0171) and 4.2 folds (*P* = 0.01) in N5D1 and N5D2, respectively (Figure 2D).

*NlKr-h1* RNAi resulted in significant mortality (higher than ds*GFP* controls, *P* = 0.0031) at 5 days after injection (Figure 3B). At the phenotypic level, the ds*GFP* group (*n* = 59 out of 60) molted to normal N5 nymphs and then adults, while the ds*NlKr-h1* injected individuals (*n* = 46 out of 60) failed to molt to normal N5 nymphs. Instead, 34 (74%) of these 46 individuals were molted into nymph–adult intermediates (adultoid) and remained as such without further molting until death in a span of 5–7 days. These nymph–adult intermediates had deformed membranous wings (not well extended) and external genitalia rudiments (Figure 4B). The other 26% individuals died of incomplete ecdysis due to parts of the exuvium could not get detached from the abdomen. These individuals also had deformed membranous wings. All of the *NlKr-h1* RNAi individuals had four carinae on the frons which is a characteristic feature of nymphs. Corresponding with no reduction of *Kr-h1* expression level, the ds*NlKr-h1* injections into the fifth-instar nymphs did not produce any precocious nymph–adult intermediates, and the insects suffered no additional mortality compared to the ds*GFP* treated individuals.

## Discussion

In the present study, we have cloned and characterized *E93* and *Kr-h1* genes in *N. lugens* and determined their expression profiles. More importantly, the RNAi approach provided direct evidence that NlE93 and NlKr-h1 are inhibitors of each other and simultaneously involved in regulating the metamorphosis of *N. lugens*. The balance between these two genes may play a critical role in determining the next molt type. We have also identified additional transcript variants of *Kr-h1* in *N. lugens*. Multiple variants of *Kr-h1* genes are found in *D. melanogaster* (Pecasse et al., 2000; Beck et al., 2004), *F. occidentalis* (Minakuchi et al., 2011), *A. aegypti* (Cui et al., 2014), and *B. mori* (Kayukawa et al., 2012). In signaling cascades, crucial signaling components are frequently associated with redundant isoforms exemplified by the MAP kinases, NF-κB inhibitors, Wnt proteins, and others, allowing the redundant isoforms to work synergistically (Kafri et al., 2009). In *B. mori*, Kr-h1 directly binds to the consensus Kr-h1 binding site that located in the *E93* promoter region to repress its transcription (*BmE93A/B*) in a cell-autonomous manner, preventing larva from bypassing the pupal stage and progressing to precocious adult development (Kayukawa et al., 2017). Similarly, *E75* isoforms, another gene involved in ecdysis process, showed functional redundancy but temporal and regional regulation, mediated steroidogenesis autoregulation, and contributed to the precise control of developmental timing in *B. mori* (Li et al., 2015, 2016). Although no *E93* isoform was identified in the present work, it is likely that *Kr-h1* isoforms were involved in the modulation of *E93* transcription in *N. lugens*.

Sequence analysis of NlE93 and NlKr-h1 revealed that both contained DNA-binding domains; NlE93 possessed a HTH-type psq DNA-binding domain, whereas NlKr-h1 had a C2H2-type Zinc finger domain. The HTH-type psq DNA-binding domain is present in the eukaryotic proteins of Pipsqueak family and seems to be structurally related to the known DNA-binding domains (Lehmann et al., 1998). *Drosophila* cell death protein E93 was found to contain a psq motif and defined as a new subgroup (the third) of psq domain proteins (Siegmund and Lehmann, 2002). E93s act through two HTH domains and probably are involved in promoting metamorphosis by transducing 20-hydroxyecdysone (20E) signaling that induces larval tissue programmed cell death and remodeling (Lee et al., 2000, 2002a,b; Liu et al., 2014, 2015). The Kr-h1 proteins contain C2H2-type Zinc finger domain to bind to DNA (Wolfe et al., 2000), RNA, and proteins (Brayer et al., 2008) for their transcription regulatory functions.

The expression patterns of *NlE93* and *NlKr-h1* revealed that decreasing *NlKr-h1* mRNA levels was correlated with increasing *NlE93* mRNA levels and vice versa. The balance between the expression levels of *NlE93* and *NlKr-h1* synchronizes with JH and ecdysone titer dynamics during insect molting and metamorphosis. Periodic surges of 20E induce molting but the type of the molt depends on JH titer (Jindra et al., 2013). Higher JH titer in hemolymph results in immature molts, but the lower JH titer induces insect metamorphosis (Belles, 2011). Our results showed that the expression pattern of *NlKr-h1* is similar to the JH titer profiles (Dai et al., 2001). *NlKr-h1* mRNA was maintained at a certain level in juvenile instar nymphs, but decreased in the last instar nymphs. Similar decreasing trend was observed in many hemimetabolan species, such as *Pyrrhocoris apterus* (Konopova et al., 2011), *B. germanica* (Lozano and Belles, 2011), and *Planococcus kraunhiae* (Vea et al., 2016). These results support that metamorphosis in hemipterans begins to be “prepared” in the penultimate, not the last nymphal instar. This could be typical of hemipterans, and perhaps paraneopterans, as in scale insects, the expression of *Kr-h1* also start to decrease before the last nymphal instar. Furthermore, the decreasing trend of *NlKr-hl* at the last instar of immatures was observed in prepupae of holometabolan species, such as *D. melanogaster* (Minakuchi et al., 2008) and *T. castaneum* (Minakuchi et al., 2009). Conversely, *NlE93* transcripts were present in the later stage of penultimate instar, but increased in the last instar nymphs, which is consistent with the action of metamorphosis. In the proposed MEKRE93 pathway of hemimetabolan insects, Kr-h1 that represses E93 expression triggers the last nymph formation at the time of the 20E peaks in late penultimate nymphal instar. On the other hand, E93 that represses Kr-h1 determines the adult fate at the beginning of the last nymphal instar (Belles and Santos, 2014). However, the upstream signals for *E93* activation are yet to be characterized.

Knockdown of *NlE93* or *NlKr-h1* increased the expression level of the other, and produced supernumerary N6 instar or precocious nymph–adult intermediates (adultoid) of *N. lugens*, respectively. *E93* knockdown induced similar phenotypes (supernumerary N7 instar and second pupa) in other insects including *B. germanica* and *T. castaneum* (Ureña et al., 2014). In the current study, ds*NlE93* injection into the fourth instar nymphs (N4) did not affect immature molt (injected individuals molted into normal N5 nymphs), but the injection adversely affected adult molt (failed to produce normal adults). Similar phenotypes (precocious adults and precocious larval-pupal transition) have been reported in *B. germanica* (Lozano and Belles, 2011) and *T. castaneum* (Minakuchi et al., 2009) after *BgKr-h1* or *TcKr-h1* were knocked down. Furthermore, removal of JH at the earlier instars led to precocious metamorphosis in *T. castaneum* (Minakuchi et al., 2008) and *B. mori* (Tan et al., 2005). However, when the ds*NlKr-h1* was injected into the fifth-instar nymphs of *N. lugens*, no precocious nymph–adult intermediates were observed. These results led us to hypothesize that NlKr-h1 inhibits the expression of *NlE93* in the juvenile instar nymphs of *N. lugens* (<N5). NlE93 not only promotes metamorphosis but also inhibits the expression of *NlKr-h1* to ensure the proper metamorphosis action taking place in the later instar nymphs.

## Conclusion

In summary, the current study characterized *E93* and *Kr-h1* genes in *N. lugens*, and revealed their roles in regulating development and metamorphosis processes through evaluations of their temporal and spatial expression patterns and gene knockdown assays. The results suggested that these two genes exhibit a mutual inhibition relationship. The balance between these two genes plays a critical role in the metamorphosis of *N. lugens*, deciding the proper timing for activating metamorphosis during the nymphal stages of *N. lugens*.

## Author Contributions

KL and SY did most of the experimental work. WW, FL, and QF collected the insects. SN participated in the manuscript writing. PW designed the study, analyzed the data, and wrote the manuscript.

## Conflict of Interest Statement

The authors declare that the research was conducted in the absence of any commercial or financial relationships that could be construed as a potential conflict of interest.

## Supplementary Material

FIGURE S1 Sequence alignment and gene structure comparison of *E93* and *Kr-h1*. **(A)** The HTH motifs of E93s from *Nilaparvate lugens* (*N_lug*), *Drosophila melanogaster* (*D_mel*, NP_652002.2), *Tribolium castaneum* (*T_cas*, KYB25180.1) *Bombyx mori* (*B_mor*, AIL29268.1), and *Blattella germanica* (*B_ger*, CCM97102.1). The domain elements are marked with straight lines. **(B)** Kr-h1s from *N. lugens* (*N. lugens*), *Rhodnius prolixus* (*R. prolisxus*, AEW22980.1), *B. germanica* (*B_ger*, CCC55948.1), *Apis mellifera* (*A.mellifera*, BAL04728.1), *T. castaneum* (*T_cas*, NP_001129235.1), *B_mori* (*Bmori*_A, NP_001171332.1 and *B. mori*_B, BAL04727.1), *Aedes aegypti* (*A_aegypti*, XP_001655162.1), *Culex quinquefasciatus* (*C_qui*, XP_001863529.1), and *D. melanogaster* (*D_ melanogaster*_A, NP_477467 and *D. melanogaster*_B, NP_477466.1). The C2H2-type Zinc finger motifs (ZnF_C2H2) are marked with filled rectangles. Amino acids with 100, >80, and >60% conservation are shaded in black, dark gray, and light gray, respectively. Gaps have been introduced to permit alignment.

FIGURE S2 The duplication of cuticular structures of *N. lugens* subjected to ds*NlE93* injection. The N6 NlE93i individuals could not molt to another supernumerary instar. Dissection revealed duplicate cuticular structures. These individuals died ultimately.

TABLE S1 Primers used for RT-PCR, dsRNA synthesis, and qRT-PCR.
