# Supplementary figures and images for: The Roles of E93 and Kr-h1 in Metamorphosis of Nilaparvata lugens
Source: Front Physiol. 2018 Nov 22;9:1677. doi: 10.3389/fphys.2018.01677 (PMC6262030; doi:10.3389/fphys.2018.01677)

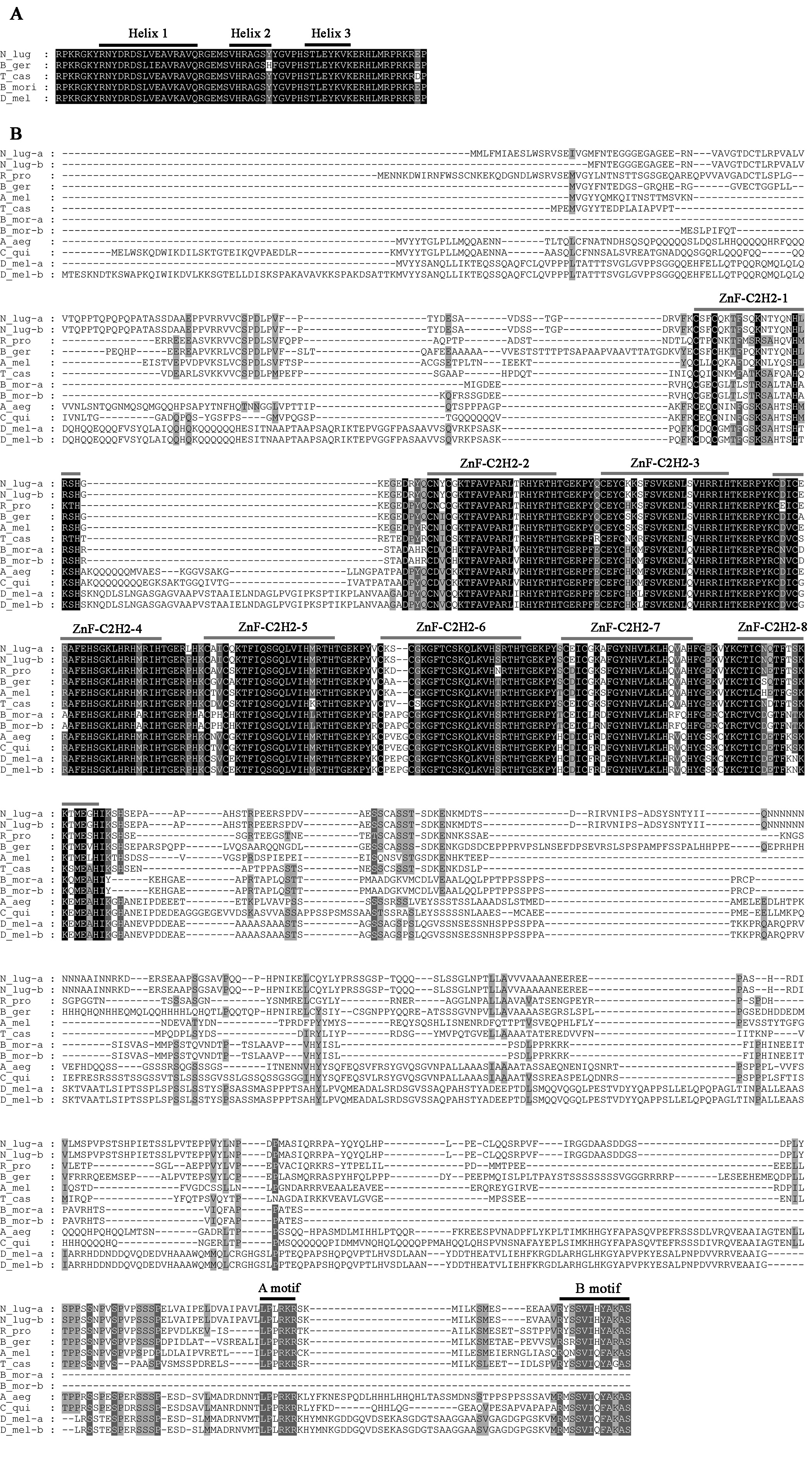

Supplement: FIGURE S1 — Sequence alignment and gene structure comparison of E93 and Kr-h1. (A) The HTH motifs of E93s from Nilaparvate lugens (N_lug), Drosophila melanogaster (D_mel, NP_652002.2), Tribolium castaneum (T_cas, KYB25180.1) Bombyx mori (B_mor, AIL29268.1), and Blattella germanica (B_ger, CCM97102.1). The domain elements are marked with straight lines. (B) Kr-h1s from N. lugens (N. lugens), Rhodnius prolixus (R. prolisxus, AEW22980.1), B. germanica (B_ger, CCC55948.1), Apis mellifera (A.mellifera, BAL04728.1), T. castaneum (T_cas, NP_001129235.1), B_mori (Bmori_A, NP_001171332.1 and B. mori_B, BAL04727.1), Aedes aegypti (A_aegypti, XP_001655162.1), Culex quinquefasciatus (C_qui, XP_001863529.1), and D. melanogaster (D_ melanogaster_A, NP_477467 and D. melanogaster_B, NP_477466.1). The C2H2-type Zinc finger motifs (ZnF_C2H2) are marked with filled rectangles. Amino acids with 100, >80, and >60% conservation are shaded in black, dark gray, and light gray, respectively. Gaps have been introduced to permit alignment. [file Image_1.JPEG]

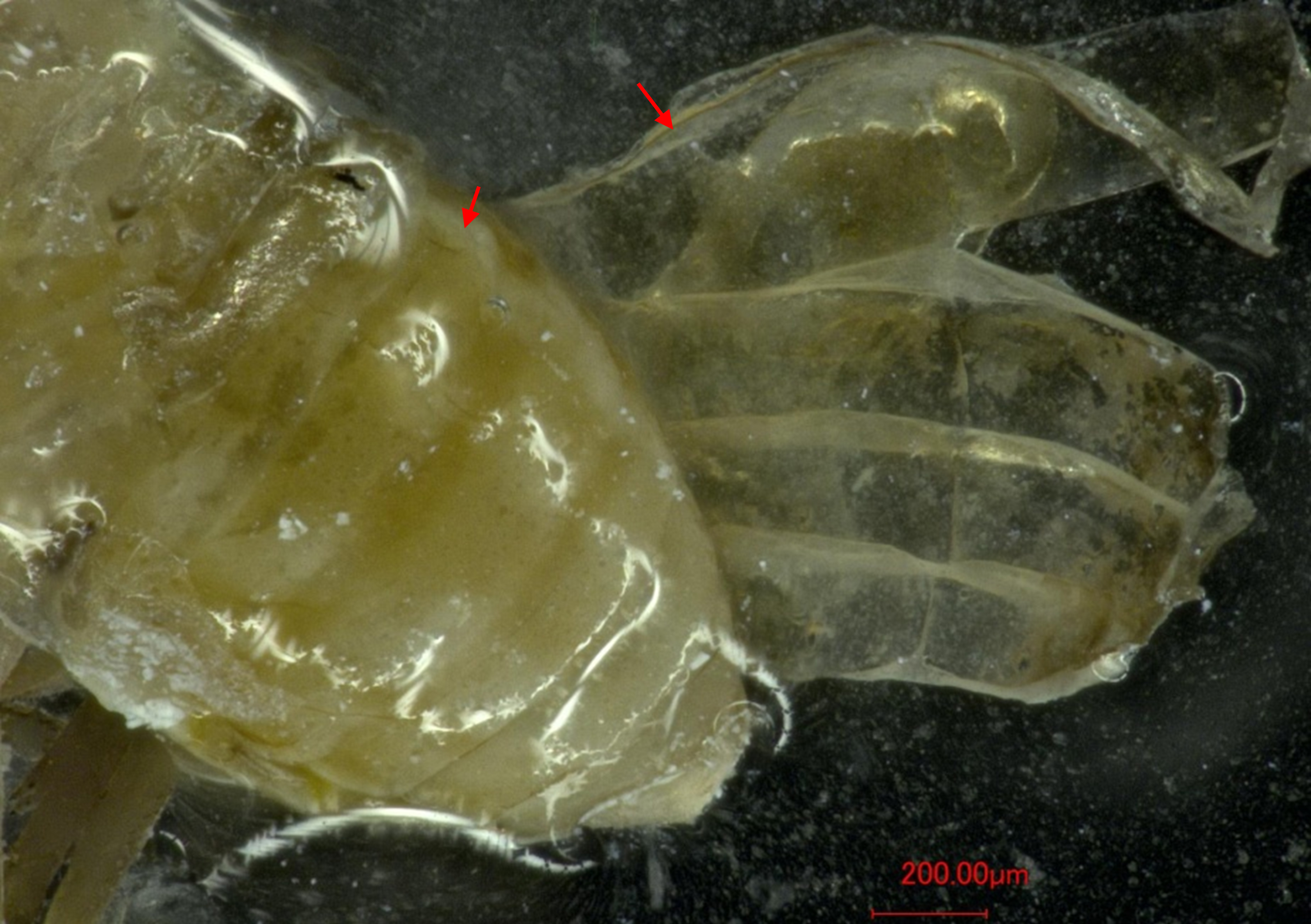

Supplement: FIGURE S2 — The duplication of cuticular structures of N. lugens subjected to dsNlE93 injection. The N6 NlE93i individuals could not molt to another supernumerary instar. Dissection revealed duplicate cuticular structures. These individuals died ultimately. [file Image_2.JPEG]
