# Supplementary material for: The Roles of E93 and Kr-h1 in Metamorphosis of Nilaparvata lugens
Source: Front Physiol. 2018 Nov 22;9:1677. doi: 10.3389/fphys.2018.01677 (PMC6262030; doi:10.3389/fphys.2018.01677)
Supplement: TABLE S1 — Primers used for RT-PCR, dsRNA synthesis, and qRT-PCR. [file Table_1.DOCX]

**Table S1. Primers used for RT-PCR, dsRNA synthesis and qRT-PCR.**

| **Primer name** | **Forward sequence (5'-3')†** | **Reverse sequence (5'-3')†** | **Amplicon size (bp)** |
| --- | --- | --- | --- |
| **RT-PCR** |  |  |  |
| *NlE93* | GGACTCGTCTCGCAGGAAAT | GTGAAGGCTGTCAATGTGCT | 3424 |
| *NlKr-h1_A* | CAAGCGCCAGTGAAAGTGAG | AGGCCGAATGTTGCATGTTG | 1985 |
| **dsRNA synthesis** |  |  |  |
| *dsNlE93* | T7-AAAACGGGGCAAGTACAGGA | T7-TCTGGGGTAGTCGACAGGAA | 433 |
| *dsNlKr-h1* | T7- CGCCAGTGAAAGTGAGACCT | T7- GAGACCGCAAGTGGTTCTGA | 498 |
| *dsGFP* | T7-CCTGAAGTTCATCTGCACCAC | T7-TGATGCCGTTCTTCTGCTTGT | 355 |
| **qPCR** |  |  |  |
| *qNlE93* | GAATGCAGTTGCCTCTTCAA | CGACGACACCAGATGATACC | 133 |
| *qNlKr-h1* | AGAAAGCGCTCCAAGATGAT | GAGGCCTTGGCATAGTGAAT | 89 |
| *qNlRPS11* | CCGATCGTGTGGCGTTGAAGGG | ATGGCCGACATTCTTCCAGGTCC | 159 |
| *qNlRPS15* | TAAAAATGGCAGACGAAGAGCCCAA | TTCCACGGTTGAAACGTCTGCG | 150 |

Note: T7 promoter sequences, 5**'**-TAATACGACTCACTATAGGGAGA-3**'**
